# Analysis by RNA-seq of transcriptomic changes elicited by heat shock in *Leishmania major*

**DOI:** 10.1038/s41598-019-43354-9

**Published:** 2019-05-06

**Authors:** Alberto Rastrojo, Laura Corvo, Rodrigo Lombraña, Jose C. Solana, Begoña Aguado, Jose M. Requena

**Affiliations:** Centro de Biología Molecular “Severo Ochoa” (CSIC-UAM), Campus de Excelencia Internacional (CEI) UAM+CSIC, Universidad Autónoma de Madrid, Madrid, Spain

**Keywords:** Microbial genetics, Transcriptomics

## Abstract

Besides their medical relevance, *Leishmania* is an adequate model for studying post-transcriptional mechanisms of gene expression. In this microorganism, mRNA degradation/stabilization mechanisms together with translational control and post-translational modifications of proteins are the major drivers of gene expression. *Leishmania* parasites develop as promastigotes in sandflies and as amastigotes in mammalians, and during host transmission, the parasite experiences a sudden temperature increase. Here, changes in the transcriptome of *Leishmania major* promastigotes after a moderate heat shock were analysed by RNA-seq. Several of the up-regulated transcripts code for heat shock proteins, other for proteins previously reported to be amastigote-specific and many for hypothetical proteins. Many of the transcripts experiencing a decrease in their steady-state levels code for transporters, proteins involved in RNA metabolism or translational factors. In addition, putative long noncoding RNAs were identified among the differentially expressed transcripts. Finally, temperature-dependent changes in the selection of the spliced leader addition sites were inferred from the RNA-seq data, and particular cases were further validated by RT-PCR and Northern blotting. This study provides new insights into the post-transcriptional mechanisms by which *Leishmania* modulate gene expression.

## Introduction

Unicellular organisms and cells in multicellular organisms, albeit to different extent, are exposed to sudden changes in the environment that compromise their viability. To cope with these stresses, microorganisms have evolved sensing mechanisms and signal transduction systems that promote adaptation, among others, in metabolism, cell cycle progression, proteostasis, cytoskeletal organization and membrane architecture^1^. Accordingly, gene expression changes are a major component of stress responses, ranging from post-translational effects, which will provide immediate responses, to regulation of gene transcription, which will be essential for the slower, long-term adaptation and recovery phases^2^.

Response to heat stress is universal, and it is being extensively studied as a model for understanding the interplay between molecular sensors, signal transduction pathways, gene expression changes and effector processes. A particularly interesting case is found in digenetic parasites that along their life cycle alternate between poikilothermic and homoeothermic hosts. In these organisms, like *Leishmania*, sudden temperature variations during transmission are natural events in their life cycles^3^. Thus, *Leishmania* parasites proliferate as flagellated promastigotes in the gut of sandflies and as amastigotes inside mammalian macrophages; transmission from the insect vector to the mammalian host includes a drastic increase over ambient temperature by more than 10 °C. In fact, the differentiation from the promastigote stage to an amastigote-like form can be induced in a host-free system by exposure promastigotes to 37 °C and an acidic pH^4,5^. Therefore, the heat shock response itself may be considered as an integral part of the developmental program in this parasite^6,7^. However, at present, the molecular mechanisms underlying the differentiation process remain poorly understood.

On the other hand, the heat shock response in *Leishmania* is distinguished from the stress response of the metazoa and yeasts by a lack of regulation at the transcription level. Thus, *Leishmania* and related trypanosomatids regulate the entire gene expression almost exclusively at the post-transcriptional level^8–10^. This feature has modelled the genome architecture of these parasites: genes are organized into large collinear clusters present on a single strand, and the different gene clusters are separated from each other by short sequences of a few kilobases (kb), or shorter (<1-kb), termed strand-switch regions (SSRs), where the transcription sense converges or diverges^11^.

Transcription initiation sites are located at the beginning of the gene clusters^12^ and a few other sites within the clusters^13^. The gene clusters are transcribed into polycistronic RNA precursors that are subsequently processed into individual mRNAs by *trans*-splicing and polyadenylation^14^. Therefore, in pursuing differential expression of individual genes, trypanosomatids have potentiated some mechanisms to control gene expression, such as differential processing of polycistronic transcripts, regulation of mRNA stability and translational activity.

Apart from its role in the differentiation program, the heat shock response in *Leishmania* is being used as a genetic model for deciphering the mechanisms regulating gene expression. Thus, studies on the expression of the two major heat shock proteins (HSPs), i.e. HSP70 and HSP83/90, contributed substantially to demonstrate that control of gene expression in this parasite occurs almost exclusively at the post-transcriptional level, and that HSP synthesis during heat shock depends on regulation of mRNA turnover and translational control^15–20^.

After sequencing of the *L. major* genome^11^, it was possible to conduct genomic-scale analysis of gene-expression changes based on the use of shotgun genome DNA or oligonucleotide microarrays. Thus, this methodology was used to detect differences in the mRNA expression levels between promastigote and amastigote stages^21–25^, changes in gene expression elicited during axenic promastigote-to-amastigote differentiation^26,27^, cadmium induced cell growth arrest^28^, metacyclogenesis^29,30^ and species-specific gene expression^31^. Nevertheless, microarrays construction and data analysis are conditioned by deficiencies in current gene annotations, in which for most genes only putative ORFs, but no UTRs, are defined. This drawback may be solved in part using microarrays constructed from cDNA clones^32^, but redundancy and under-representation of cDNAs limit their usefulness. Advances in sequencing technologies (next-generation sequencing or NGS), and particularly their application to determine the sequence of all expressed RNAs (RNA-seq), and their relative abundance, has emerged as a powerful tool for determining any transcriptome without prior assumptions about the transcribed regions^33^. Hence, as a first step for analysing changes in mRNA levels, we used the RNA-seq methodology to establish a comprehensive poly-A transcriptome for the promastigote stage of *L. major*^34^. A total of 10,285 transcripts were identified, of which 1,884 did not match with previously annotated genes and therefore were categorized as novel genes. Moreover, this analysis generated structural information (i.e. delimitation of ORFs, and 5′- and 3′-UTRs) for most of the genes, together with accurate estimations on the relative abundance of transcripts^34^. Based on the established *L. major* transcriptome, in the present work we have analysed by RNA-seq the transcript expression changes triggered by a short incubation of promastigotes at 37 °C. Significant differences in the mRNA levels were detected for a third of the transcripts, indicating that temperature increase have a marked effect on the *L. major* transcriptome. As a result, we are providing a comprehensive list of heat shock regulated genes, including many coding for previously unstudied proteins and even putative non-coding RNAs. Also, alternative *trans-*splicing was envisaged as an additional mechanism involved in differential gene expression in *Leishmania*.

## Results and Discussion

### Identification of differentially expressed transcripts by RNA-seq

The life cycle of *Leishmania* includes a transmission from poikilothermic phlebotomine insects into the homeothermic mammalian host. Thus, during transmission, elevated temperature is the first signal encountered by the parasite upon its entry into the mammalian host. However, as a regular feature of the parasite’s life cycle, this abrupt change in temperature (from environmental temperature to 34–37 °C) does not represent a severe heat shock for the parasite, and the general processes of transcription and translation are not substantially affected^18,35^. Nevertheless, changes in the expression at the level of RNA or/and protein abundances have been observed for particular genes when promastigotes are incubated at 37 °C (reviewed in^36^).

**Figure 1:**
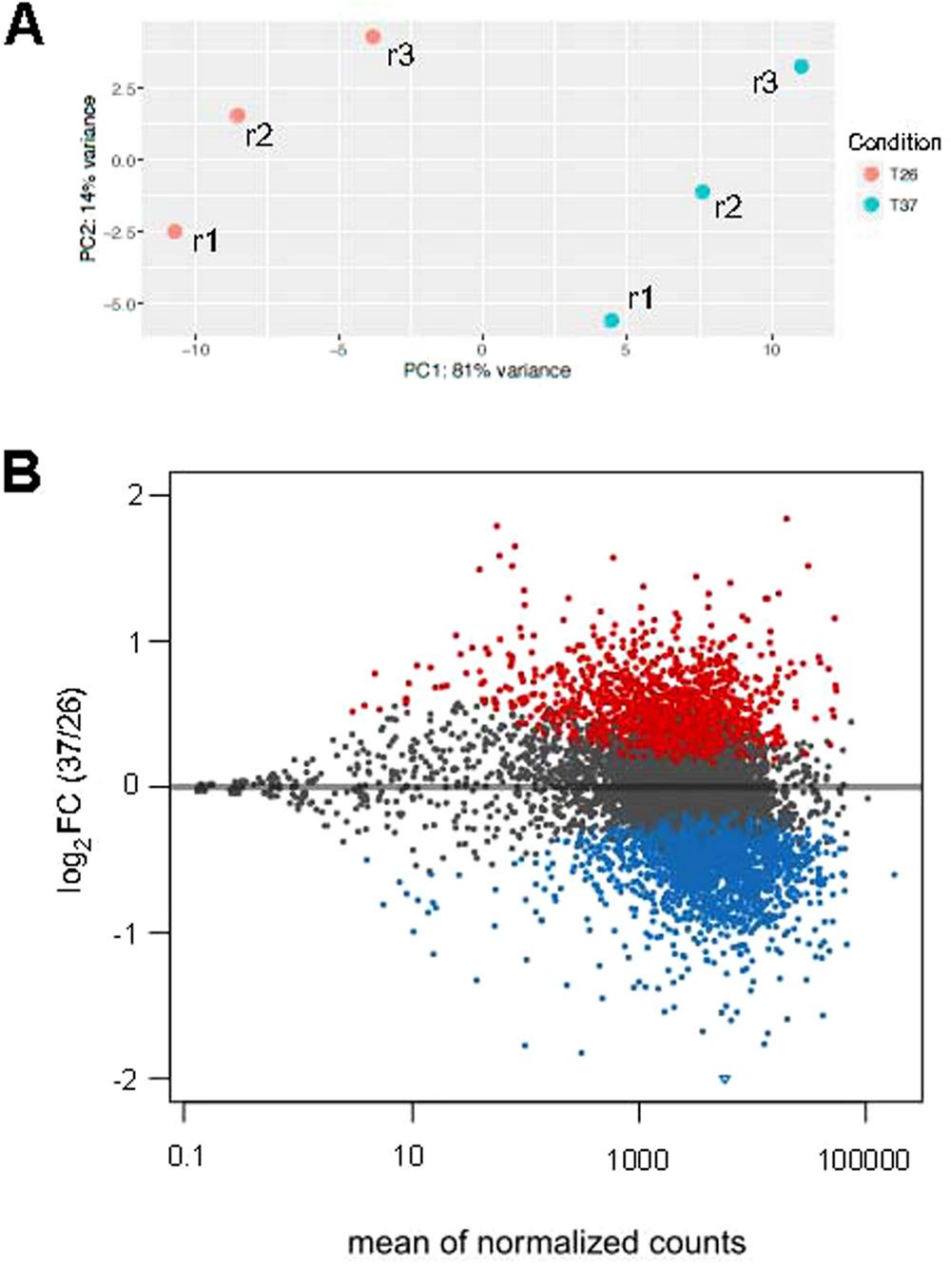
Analysis of the global transcriptional changes, determined by RNA-seq, during heat shock in *L. major* promastigotes. (**A**) PCA plot of the three replicates (r1–r3) derived from each experimental condition (26 or 37 °C). (**B**) Differential expression analysis of the 10,700 transcripts annotated in the *L. major* genome. The plot shows the relationship between the mean of the normalized counts and the fold change (log_2_) between 26 and 37 °C conditions. Each point represents one transcript. Points colored in red or blue represent transcripts significantly up- or down-expressed at 37 °C, respectively; points colored in black correspond to transcript showing a constant expression in both conditions.

In this work, we have used RNA-seq to analyse changes in the *L. major* transcriptome promoted by incubation of promastigotes for two hours at 37 °C. Total RNA from the six samples (three cultures grown at 26 °C and the matched cultures incubated for 2 h at 37 °C) was extracted and after poly(A)^+^ selection, RNA sequencing was carried out using the Illumina methodology (see Materials and Methods for further details). In previous works, we annotated the poly-A transcriptome based on the *L. major* promastigote form^34^ and improved its reference genome^37^. The *L. major* transcriptome was modified accordingly and the current version is available at Leish-ESP web server (leish-esp.cbm.uam.es). Table 1 summarizes the RNA-Seq datasets generated for this study. Principal component analysis (PCA) was used to analyse the relationship between samples (Fig. 1A), showing a clear separation between the samples derived from promastigotes incubated at 26 °C or 37 °C and excluding that experimental variations (‘batch effect’) added unwanted variability into the study.

**Table 1:** Statistics for RNA-Seq data sets.

Replicate (T)	Reads (2 × 76 bp)	% aligned reads^a^	SL-reads	% SL-reads
1 (26 °C)	18,545,439	96.18	687,928	3.71
2 (26 °C)	37,465,333	96.29	1,227,685	3.28
3 (26 °C)	32,304,297	96.50	1,092,996	3.38
1 (37 °C)	26,510,688	96.48	851,959	3.21
2 (37 °C)	32,684,273	96.56	1,054,854	3.23
3 (37 °C)	25,634,781	96.53	794,935	3.10

^a^Reads alignment was done by Bowtie2 using the updated *L. major* (Friedlin) genome^37^.

To compare the expression levels between promastigotes growth at 26 °C and after heat shock, the numbers of reads mapping within each one of the *L. major* annotated transcripts^34^ (also available at Leish-ESP web server) were determined. The analysis of relative expression levels of the transcripts was done by the DESeq2 program. Out of the 10,700 transcripts currently annotated in the *L. major* nuclear genome, 1,866 (18%) were found to be significantly upregulated and 2,103 (20%) downregulated at 37 °C, regarding the expression levels at 26 °C. Figure 1B shows a scatter plot in which all the transcripts were plotted according to the mean of counts per transcript after normalization (mean of normalized counts) and the fold change (FC) for each transcript at 37 °C versus 26 °C. See also Supplementary Dataset 1 for the complete list of transcripts, showing the individual expression levels together with their FC values associated with the temperature treatment.

**Figure 2:**
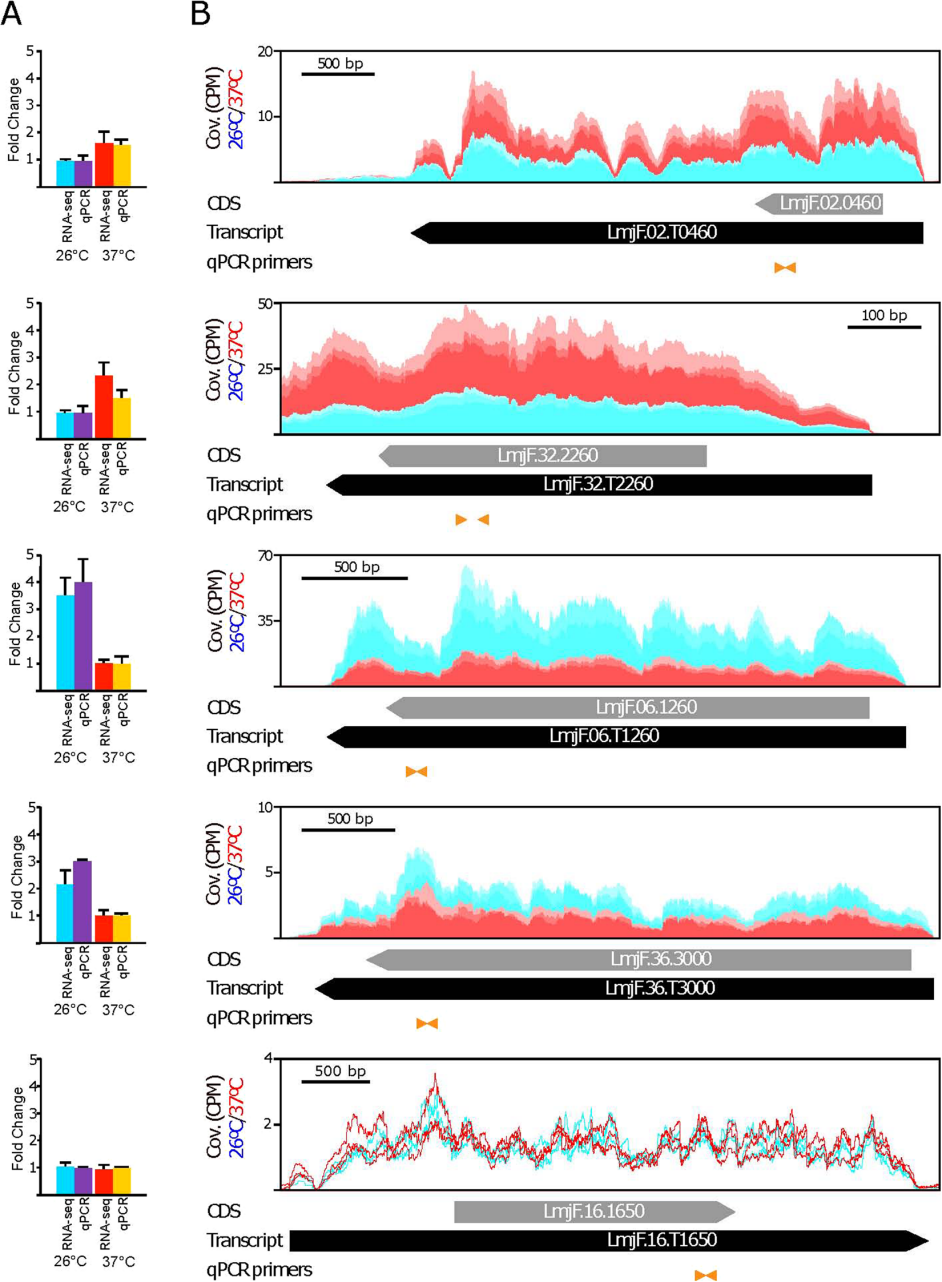
Comparative analysis of the relative expression levels of selected transcripts determined by RNA-seq and validated by quantitative RT-PCR (qPCR). Based on the RNA-seq analysis, two up-regulated transcripts (LmjF.02.T0460 and LmjF.32.T2260), two down-regulated (LmjF.06.T1260 and LmF.36.T3000) and one that did not change (LmjF.16.T1650) after the temperature treatment of the parasites were selected for validation by real-time PCR. In (**A**) fold-change values in the transcript levels between normal (26 °C) and heat shocked (37 °C) parasites determined by RNA-seq and qPCR. For comparison, and for each one of the transcripts, the lowest values were set arbitrary as 1. The arithmetic mean from triplicate cultures and the standard deviation are represented. In (**B**) RNA-seq reads derived from promastigotes incubated either at 26 °C (blue) or 37 °C (red), three replicates each, were mapped independently on the genomic region containing the specific transcript (black arrow; the CDS location is represented by a grey arrow). The positions of the oligonucleotides used for qPCR determinations are shown by orange arrowheads. Coverage (Cov.) is expressed as counts per million of reads (CPM).

Although RNA-seq is found to be a highly reproducible technique to quantify transcript levels^38^, we decided to carry out a validation by real time-PCR (qRT-PCR). For this purpose, we selected two upregulated transcripts (LmjF.02.T0460 and LmjF.32.T2260), two downregulated transcripts (LmjF.06.T1260 and LmjF.36.T3000) and one invariant transcript (LmjF.16.T1650). As shown in Fig. 2, agreement between the RNA-seq and qRT-PCR data was observed for all the analyzed transcripts.

**Figure 3:**
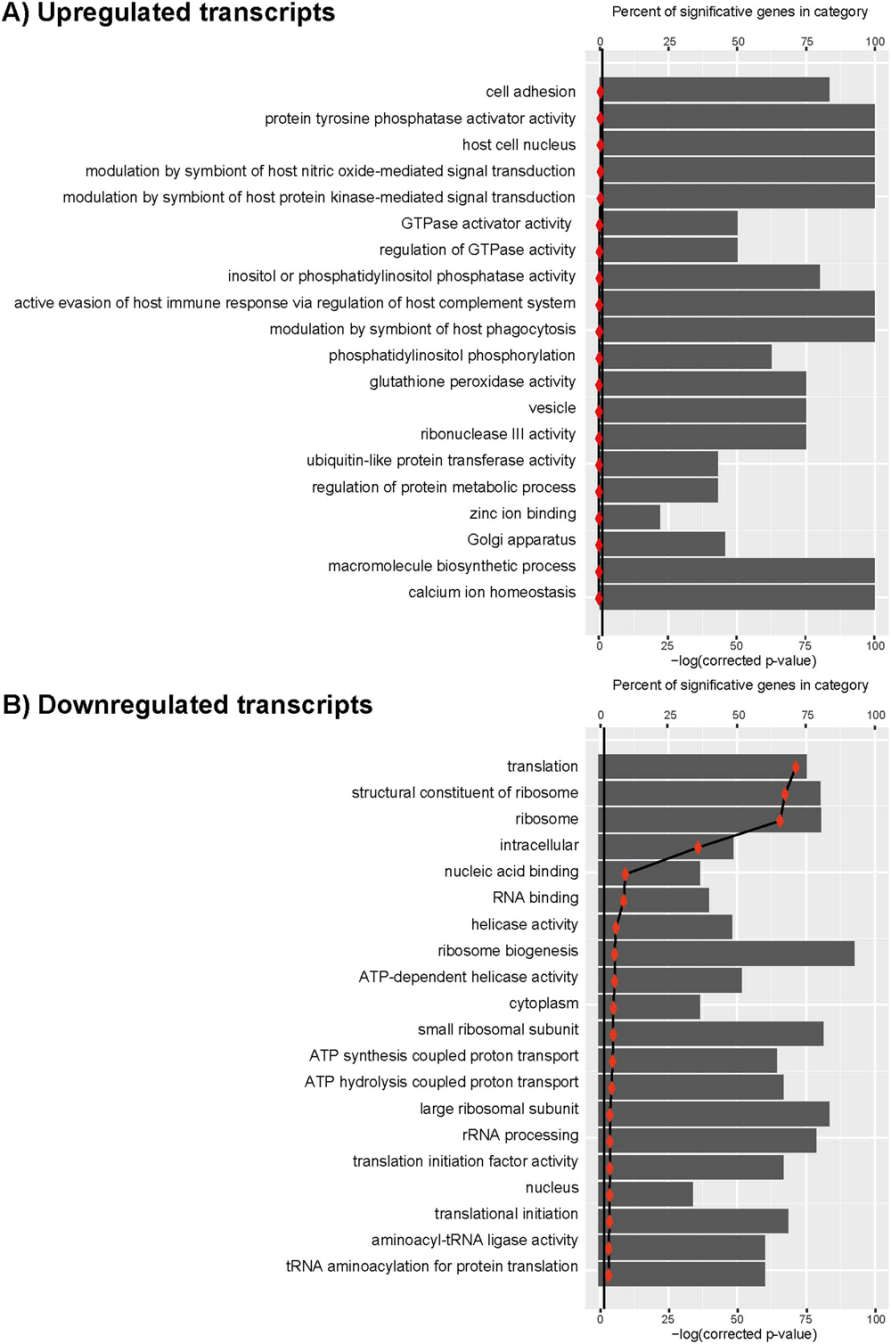
Gene Ontology (GO) analysis of upregulated (panel A) and downregulated (panel B) transcripts. GO terms associated to annotated genes were extracted from the *L. major* (Friedlin strain) database and then mapped to the corresponding transcript in the transcriptome (Supplementary Dataset 3). This information was then loaded together with DESeq2 output into goseq R package to carry out GO enrichment analysis. Vertical line marks the negative logarithm for a p-value = 0.05.

The lists of transcripts showing different expression levels were used to define enriched gene ontology (GO) categories (Fig. 3). Using the upregulated transcripts, none of the most represented GO categories were found to be significantly enriched (Fig. 3A). However, twenty GO categories were identified as significantly enriched (corrected P-value lower than 0.05) using the downregulated transcripts (Fig. 3B). The more enriched GO categories were those related to translation and ribosome structure. Although useful, GO enrichment analysis should be considered with caution. Among downregulated transcripts, only 961 have known functions, 867 are annotated as hypothetical proteins and 275 are unknown. In total, only 1,113 transcripts out of 2,103 (53%) have an associated GO. On the other hand, among the 1,866 upregulated transcripts, 1418 have annotated ORFs, but only 634 (34%) have an associated GO term; the rest (784) of the annotated ORFs are defined as hypothetical proteins.

Taking into account the large number of transcripts whose expression levels resulted significantly altered by the temperature treatment, we decided to pay special attention on those transcripts whose levels showed changes above 2-fold.

### Transcripts with annotated ORFs whose levels increased after heat shock

A total of 52 transcripts were found to be up-regulated (2-fold or more) after incubation of *L. major* promastigotes at 37 °C for 2 h (Table 2). Among them, 33 transcripts corresponded to annotated protein-coding genes (even though 20 are annotated as coding for hypothetical proteins), one structural RNA (small nuclear RNA, U2 snRNA), and 18 transcripts lacked annotation in the GeneDB database (those transcripts are indicated as “unknown” in Table 2).

**Table 2:** Transcripts UP-regulated after 2 h at 37 °C.

Transcript_ID^a^	log_2_FC(37/26)	Reference gene^b^	Description^c^
**Protein folding/stress response**			
LmjF.34.T0070	1.839	LmjF.34.0070	ascorbate peroxidase (APX)
LmjF.29.T2450	1.327	LmjF.29.2450	heat shock protein 20
LmjF.33.T0365	1.295	LmjF.33.0365	HSP83/90
LmjF.32.T2260	1.291	LmjF.32.2260	heat shock protein Hsp20
LmjF.29.T1270	1.291	LmjF.29.1270	HSP100
LmjF.28.T2780	1.156	LmjF.28.2780	HSP70 (type-I transcript)
LmjF.33.T0318	1.091	LmjF.33.0318	HSP83/90
**Nucleosome assembly**			
LmjF.17.T1220	1.442	LmjF.17.1220	histone H2B
LmjF.21.T0930	1.070	LmjF.21.0930	histone H2A
**Other functions**			
LmjF.28.T1570	1.232	LmjF.28.1570	hydrolase, alpha/beta fold family, putative
LmjF.17.T1010	1.017	LmjF.17.1010	hydrolase, alpha/beta fold family-like protein
LmjF.19.T1347	1.014	LmjF.19.1347	membrane-bound O-acyltransferase, putative
LmjF.08.T0700	1.003	LmjF.08.0700	amastin-like protein
**Hypothetical proteins**			
LmjF.35.T2600	1.791	LmjF.35.2600	hypothetical protein, unknown function
LmjF.35.T2610	1.591	LmjF.35.2610	hypothetical protein, unknown function
LmjF.08.T0860	1.515	LmjF.08.0860	hypothetical protein, unknown function
LmjF.23.T1665	1.399	LmjF.23.1665	hypothetical protein
LmjF.08.T1225	1.373	LmjF.08.1225	hypothetical protein, unknown function
LmjF.05.T0810	1.190	LmjF.05.0810	hypothetical protein, conserved
LmjF.29.T2070	1.156	LmjF.29.2070	hypothetical protein, unknown function
LmjF.31.T0120	1.145	LmjF.31.0120	hypothetical protein, conserved
LmjF.27.T1740	1.145	LmjF.27.1740	hypothetical protein, unknown function
LmjF.12.T1050	1.143	LmjF.12.1050	hypothetical protein, conserved
LmjF.08.T1270	1.122	LmjF.08.1270	hypothetical protein (amidinotransferase domain)
LmjF.12.T0800	1.111	LmjF.12.0800	hypothetical protein, conserved
LmjF.13.T0590	1.107	LmjF.13.0590	hypothetical protein, conserved
LmjF.07.T0745	1.105	LmjF.07.0745	hypothetical protein
LmjF.35.T4240	1.066	LmjF.35.4240	hypothetical protein, conserved
LmjF.12.T0840	1.041	LmjF.12.0840	hypothetical protein, conserved
LmjF.17.T0860	1.029	LmjF.17.0860	hypothetical protein, unknown function
LmjF.36.T4050	1.021	LmjF.36.4050	hypothetical protein, conserved (TatD related DNase domain)
LmjF.28.T1120	1.016	LmjF.28.1120	hypothetical protein, conserved
LmjF.12.T1030	1.012	LmjF.12.1030	hypothetical protein, conserved
**Transcripts lacking protein-coding annotation**			
LmjF.35.T0095	1.651	—	unknown
LmjF.32.T2675	1.573	—	unknown
LmjF.15.T0432	1.503	—	unknown
LmjF.29.T1832	1.351	—	unknown
LmjF.29.T1265	1.324	—	unknown
LmjF.10.T0605	1.249	—	unknown
LmjF.36.T2285	1.232	—	unknown
LmjF.27.T0215	1.203	—	unknown (ORF coding for 117 amino acids)
LmjF.01.T0795	1.169	—	unknown (ORF coding for 112 amino acids)
LmjF.33.T0537	1.097	—	unknown
LmjF.19.T0595	1.072	—	unknown
LmjF.36.T5945	1.071	—	unknown
LmjF.32.T3345	1.058	—	unknown (ORF coding for 93 amino acids)
LmjF.22.T1625	1.052	—	unknown
LmjF.26.T0465	1.040	—	unknown
LmjF.31.TsnRNA.01	1.039	LmjF.31.snRNA.01	small nuclear RNA, U2 snRNA
LmjF.23.T1595	1.034	—	unknown
LmjF.02.T0075	1.022	—	unknown
LmjF.08.T1237	1.014	—	unknown

^a^Transcript identifier (ID) according to Rastrojo *et al*.^34^. Additional information is available at Leish-ESP server (http://leish-esp.cbm.uam.es/).

^b^Gene ID according to GeneDB database.

^c^Hypothetical protein: predicted bioinformatically. Conserved: predicted protein of unknown function that is also annotated in *T. brucei* and/or *T. cruzi* genomes (GeneDB.org). Unknown: non-demonstrated protein-coding function.

For the transcripts containing annotated genes, the most upregulated one was LmjF.34.T0070, coding for the ascorbate peroxidase APX. Saxena and co-workers^26^, analysing changes in RNA abundance during axenic promastigote-to- amastigote differentiation in *L. donovani*, also found increased levels of this transcript after 5 h of exposure to the differentiation stimulus (37 °C in acidic medium). Similarly, in a recent study, the APX, at both transcript and protein levels, revealed ~5-fold upregulation in axenic amastigotes compared to promastigotes^39^. The kinetics properties of this enzyme in *L. major* have been characterized^40^. This peroxidase may play an important role in detoxification of H_2_O_2_, which is generated during the oxidative burst of infected host macrophages. Remarkably, the APX gene was found to be overexpressed in *L. donovani* clinical isolates that are resistant to Amphotericin B, a first-line drug for treatment of visceral leishmaniasis^41^.

**Figure 4:**
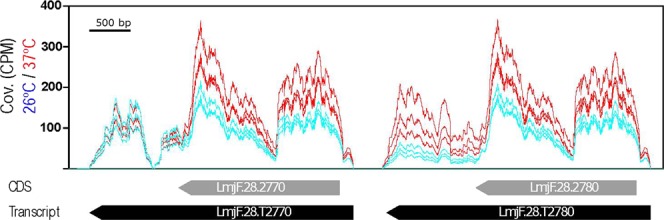
Temperature-dependent expression levels for the transcripts derived from the *HSP70* locus. Alignment on the *L. major HSP70* gene locus of the RNA-seq reads obtained from RNA samples (three biological replicates) isolated from either promastigotes growth at 26 °C (blue) or heat shocked at 37 °C (red). The position of the two transcripts, LmjF.28.T2270 and LmjF.28.T2280 (black arrows), transcribed from the *HSP70* locus, is indicated together with the location of their corresponding CDS (grey arrows). Coverage (Cov.) is expressed as counts per million of reads (CPM).

Several of the upregulated transcripts code for heat shock proteins (Table 2). This was an expected result, since previous studies documented the temperature-dependent accumulation for some of those transcripts (reviewed in^42^). Thus, regarding gene expression, the best studied HSP gene is that coding for the prototypical HSP70. This protein is encoded by two types of genes, *HSP70-I* (LmjF.28.2780) and *HSP70-II* (LmjF.28.2770), which are tandemly linked in the same chromosomal locus^43^. However, only transcripts derived from *HSP70-I* genes were found to increase during heat-shock. Interestingly, our RNA-seq data indicated that transcript LmjF.28.T2780 (*HSP70-I*) is 2.2-fold more abundant at 37 °C than at 26 °C while the levels of transcript LmjF.28.T2770 were not affected by the heat shock treatment (Fig. 4), in agreement with the expression levels determined by Northern blotting^43^. As illustrated in the figure, the relative expression of both transcripts can be quantified only from counting the reads mapping at the divergent 3′UTRs, as coding regions and 5′UTRs are identical in both genes. When a given read can be aligned to two or more genomic regions, the aligners distribute randomly the reads among the target regions. This explains that a similar coverage is observed in the ORFs of both transcripts, giving the false insight that the levels of both transcripts increased after heat shock. However, looking at the 3′-UTRs, the reads coverage in this region of transcript LmjF.28.T2780 did not change after heat treatment of promastigotes, indicating that the steady state levels of this transcript is not affected by heat shock, in agreement with previous data^43^. These results invoke two relevant issues that should be seriously considered when gene expression levels are measured from RNA-seq data: first, a correct annotation of the transcriptome needs to be generated; second, mapping of reads only on the ORF should be avoided when repeated genes are considered, and *Leishmania* genomes contain many repeated genes^10^.

Another evolutionary, highly-conserved heat shock protein is HSP90, which in *Leishmania* and other organisms is often named HSP83^36^. In the *L. major* genome database, 17 HSP83 genes are annotated (from gene LmjF.33.0312 to gene LmjF.33.0365) that are tandemly ordered in a single locus. Temperature-dependent increase in the *HSP83/90* mRNA levels was reported in promastigotes from both *L. amazonensis*^44^ and *L. infantum*^18^. In our study, two HSP83/90 transcripts (LmjF.33.T0365 and LmjF.33.T0318) were found upregulated more than 2-fold in promastigotes incubated at 37 °C (Table 2).

Transcript LmjF.29.T1270, which encodes for HSP100 (the homologue to the bacterial ClpB gene and to the yeast Hsp104 gene^42^) was found to be 2.45-fold more abundant in promastigotes incubated at 37 °C than in those grown at 26 °C (Table 2); this is in agreement with previous studies by Northern blotting^45^. Additionally, the levels of transcripts LmjF.29.T2450 and LmjF.32.T2260, coding for proteins belonging to the family of small HSPs^42^, increased above 2-fold after heat shock (Table 2). There are not previous studies addressing the expression levels of these transcripts; however, the protein encoded by transcript LmjF.32.T2260 is one of the five proteins that were described as strongly (or exclusively) phosphorylated in axenic amastigotes^46^.

**Figure 5:**
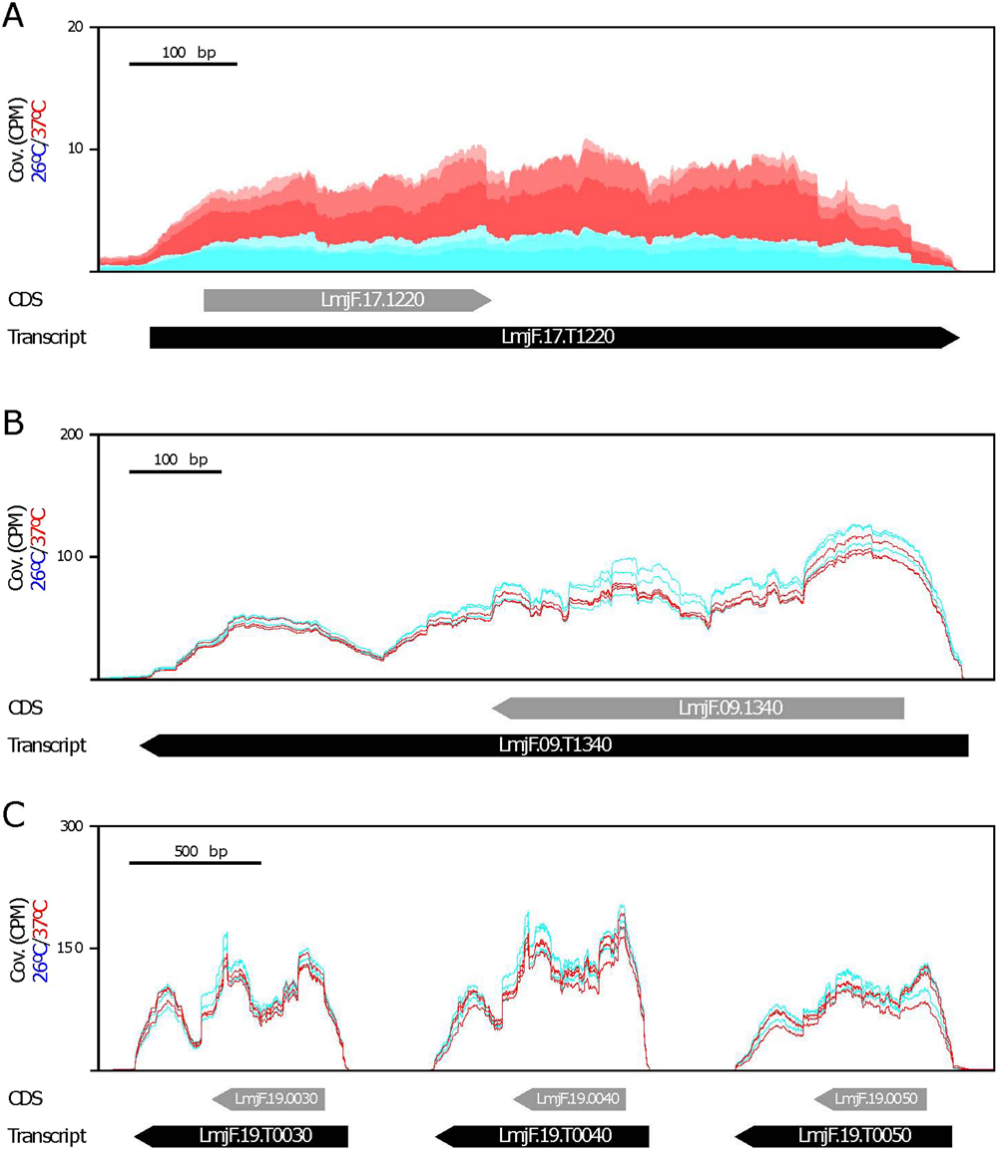
Expression levels for the histone H2B transcripts. There exist three H2B loci in the *L. major* genome: (**A**) LmjF.17.1220 gene, (**B**) LmjF.09.1340 gene, and (**C**) LmjF.19.0030, LmjF.19.0040 and LmjF.19.0050 genes. Alignment of the RNA-seq reads derived from RNA samples (three biological replicates) isolated from either promastigotes growth at 26 °C (blue) or heat shocked at 37 °C (red). The location of transcripts are indicated by black arrows, whereas the location of CDS is shown by grey arrows. Coverage (Cov.) is expressed as counts per million of reads (CPM).

Another remarkable finding was the presence of a transcript coding for the histone H2B (LmjF.17.T1220). Current annotation of the *L. major* genome indicates the presence of five H2B coding genes: LmjF.09.1340, LmjF.17.1220 and the tandemly linked genes LmjF.19.0030-0040-0050. Figure 5 shows the distribution of RNA-seq reads derived from the two sets of RNA samples in the three H2B loci. The coverages generated from the two sets of RNA-seq reads are similar for all H2B transcripts, except for transcript LmjF.17.T1220, in which the transcript levels increased at 37 °C (Fig. 5A). Interestingly, in the proteomics analysis carried out by the Zilberstein’s group^47^, the H2B protein encoded by gene LinJ.17.1320, which is the orthologue to gene LmjF.17.1220, was found to accumulate quickly after incubation of *L. donovani* promastigotes at 37 °C in an amastigote differentiation medium. Remarkably, the protein encoded by gene LmjF.17.1220 is the most divergent among the *Leishmania* H2B histones and, presumably, it might be playing a peculiar role in the chromatin architecture and consequently be affecting global transcriptional activity.

Similarly, a transcript coding for histone H2A, LmjF.21.T0930, was found to be 2.1-fold more abundant in temperature-treated promastigotes (Table 2). In the *L. major* (Friedlin) genome, there are six genes coding for this histone, grouped in two clusters with three genes each. However, only the expression levels of transcript LmjF.21.T0930 were found to be affected by heat shock (see Supplementary Dataset 1). Interestingly, the overexpression of histone H2A has been linked to an increased resistance of *L. donovani* to antimonials and other antileishmanial drugs, pointing to a possible role of this protein in overcoming the drug-elicited stress^48^. Also, the *L. infantum* orthologous protein (encoded by gene LinJ.21.1170) was found to be enriched in the membrane of axenic amastigotes^49,50^.

Other upregulated transcripts for which there exist published data about expression (protein and/or RNA levels) are discussed briefly hereafter. For transcript LmjF.28.T1570, which is annotated as coding for a putative hydrolase of the alpha/beta fold family, exists evidence in *L. infantum* that the orthologous protein (LinJ.28.1700) accumulates quickly (2.5 h) after incubating promastigotes at 37 °C in an amastigote differentiation medium^47^. Also, this protein was found in membrane-enriched fractions of *L. infantum* axenic amastigotes^49^. Additionally, this gene was identified, using oligonucleotide microarrays, as upregulated in *L. infantum* intracellular amastigotes^25^ and axenic amastigotes^51^ regarding the expression levels present in promastigotes. In Table 2, there is another transcript (LmjF.17.T1010) which is also annotated as coding for a hydrolase of the alpha/beta fold family. However, the sequence identity between LmjF.28.1570 and LmjF.17.1010 is only 42%. In agreement with our results, the transcript derived from the orthologue gene in *L. infantum* (i.e., LinJ.17.1110) was found to be 1.7-fold more abundant in intracellular amastigotes than in promastigotes^25^.

Transcript LmjF.19.T1347 (coding for a putative membrane-bound O-acyltransferase) is present in the list of genes with increased expression in *L. major* lesion-derived amastigotes^25^. Finally, among the heat shock up-regulated transcripts, several transcripts coding for members of the amastin superfamily were identified (see Supplementary Dataset 1), even though only transcript LmjF.08.T0700 showed a FC greater than 2 (Table 2). Amastin genes were first identified in *Trypanosoma cruzi*^52^, a parasite, evolutionarily related to *Leishmania*, that causes Chagas’ disease. The gene was identified due to its prominent expression in the amastigote stage regarding its expression in the epimastigote, insect-living form. Subsequently, the existence in *Leishmania* of a gene family encoding a homologue of *T. cruzi* amastin was uncovered by Papadopoulou and co-workers after a differential hybridization screening of a genomic library with cDNA probes derived from promastigote and amastigote RNAs^53^. This group carried out a detailed study about the number, sequence features, and genomic organization of this gene family in *L. infantum* and *L. major*^54^. Thus, a bioinformatics search on the *L. major* and *L. infantum* sequencing projects, allowed these authors to identify 55 *L. major* and 37 *L. infantum* amastin genes. Additionally, the authors evaluated the relative abundance of twenty amastin different transcripts in promastigotes and amastigotes of *L. infantum*; of them, 17 were found to be upregulated in axenic amastigotes^54^.

Among the up-regulated transcripts showing FC > 2, many (20 out of the 52 transcripts; Table 2) code for hypothetical proteins. Nevertheless, we made a literature search for studies in which any of these genes/proteins had been reported as differentially expressed. Thus, it was found that the *L. infantum* orthologue (LinJ.08.0800) to the transcript LmjF.08.T0860 was described as differential expressed (1.8-fold) in intracellular amastigotes^25^ and in *L. infantum* axenic amastigotes when compared to its expression in promastigotes^51^. The level of the encoded protein also increased after incubation of *L. donovani* promastigotes in conditions promoting their differentiation to axenic amastigotes^47^, and it was also found to be upregulated in *L. infantum* axenic amastigotes^55^. Similarly, an accumulation of the protein orthologue (LinJ.08.1220) to that encoded by transcript LmjF.08.T1225 was observed after incubation of *L. donovani* promastigotes in axenic conditions^47^. Transcripts LmjF.05.T0810, LmjF.013.T0590 and LmjF.19.T1347 are represented in the list of genes with increased expression in *L. major* lesion-derived amastigotes^25^. The orthologues to transcripts LmjF.31.T0120 (LinJ.31.0130) and LmjF.28.T1220 (LinJ.28.1220) showed higher expression levels in both *L. infantum* intracellular amastigotes^25^ and axenic amastigotes^51^ than in promastigotes. Also, the orthologues to transcripts LmjF.19.T1347 (LinJ.19.1370) and LmjF.35.T4240 (LinJ.35.4310) were found to be more abundant in *L. infantum* axenic amastigotes than in promastigotes^51^.

### Novel transcripts (lacking gene-annotation in current *L. major* databases) with increased expression during heat shock

High-throughput sequencing of mRNA (RNA-seq) has revealed the existence of many novel transcripts in all the organisms studied. In a previous work, our group showed that the poly-A transcriptome of *L. major* is about 20% broader than predicted by bioinformatics ORF-annotation on the genome sequence^34^. In the present work, the expression levels for 18 of the novel transcripts increased above 2-fold in promastigotes incubated at 37 °C (Table 2, transcripts without GeneDB identifier). A search for ORF indicated that only three transcripts have putative ORF larger than 90 codons: LmjF.27.T0215 (potential polypeptide of 117 amino acids), LmjF.01.T0795 (112 amino acids) and LmjF.32.T3345 (93 amino acids). To ascertain whether these putative proteins indeed exist, extensive mass spectrometry raw data of the *L. major* proteome (and other *Leishmania* species) should be generated. This approach was followed by Pawar *et al*.^56^, who showed that some of the novel RNAs reported by us in the *L. major* promastigote are indeed protein coding mRNAs. Additionally, *in situ* tagging of these putative novel proteins would represent another strategy to determine its presence and cellular localization^57^.

On the other hand, although all the novel transcripts present the miniexon sequence at the 5′ end, many might be representing long non-coding RNAs (lncRNAs). It should be noted that mammalian lncRNAs share many features with mRNAs: they are frequently transcribed by RNA polymerase II and are generally spliced, 5′ capped, and polyadenylated^58^.

### Transcripts that are down-regulated by heat shock

Table 3 lists the transcripts whose levels decreased two-fold or more in promastigotes incubated at 37 °C regarding the levels present in parasites growing at normal conditions (26 °C). Remarkably, after grouping transcripts according to GO terms, most of them could be grouped into three functional categories: transmembrane transporters, nucleic acid binding and protein translation. Also, several transcripts coding for hypothetical proteins or lacking annotated ORFs were identified as down-regulated.

**Table 3:** Transcripts down-regulated during heat-shock.

Transcript_ID^a^	log_2_(37/26)	Reference^b^	Description^c^
**Transmembrane transporters**			
LmjF.29.T0620	−1,762	LmjF.29.0620	ATP-binding cassette protein subfamily A, member 10, putative (ABCA10)
LmjF.06.T1260	−1,567	LmjF.06.1260	pteridine transporter, putative
LmjF.31.T0880	−1,502	LmjF.31.0880	amino acid permease 3 (AAP3)
LmjF.10.T0020	−1,375	LmjF.10.0020	pteridin transporter, putative
LmjF.10.T1310	−1,337	LmjF.10.1310	pteridine transporter, putative
LmjF.27.T1580	−1,335	LmjF.27.1580	amino acid transporter, putative (AAT24)
LmjF.31.T0340	−1,206	LmjF.31.0340	amino acid transporter aATP11, putative (AAT1.3)
LmjF.24.T0630	−1,192	LmjF.24.0630	ATPase subunit 9, putative
LmjF.31.T3070	−1,192	LmjF.31.3070	iron/zinc transporter protein-like protein
LmjF.11.T0660	−1,149	LmjF.11.0660	protein associated with differentiation 4, putative
LmjF.19.T0210	−1,137	LmjF.19.0210	ADP, ATP carrier protein 1, mitochondrial precursor, putative (ANC2)
LmjF.31.T0350	−1,079	LmjF.31.0350	amino acid transporter aATP11, putative (AAT1.4)
LmjF.35.T5350	−1,056	LmjF.35.5350	amino acid permease, putative (AAT27.1)
LmjF.31.T0580	−1,053	LmjF.31.0580	amino acid transporter aATP11, putative (AAT25.2)
**Nucleic acid binding**			
LmjF.05.T0140	−1,600	LmjF.05.0140	nucleolar RNA helicase II, putative
LmjF.36.T1640	−1,591	LmjF.36.1640	universal minicircle sequence binding protein (UMSBP), putative (HEXBP)
LmjF.32.T0840	−1,246	LmjF.32.0840	DRBD18, RNA binding protein
LmjF.35.T3100	−1,174	LmjF.35.3100	ATP-dependent RNA helicase, putative
LmjF.36.T1610	−1,164	LmjF.36.1610	universal minicircle sequence binding protein, putative (UMSBP1)
LmjF.26.T2630	−1,144	LmjF.26.2630	CSL zinc finger, putative
LmjF.07.T0870	−1,139	LmjF.07.0870	splicing factor ptsr1-like protein
LmjF.36.T3070	−1,095	LmjF.36.3070	fibrillarin
LmjF.35.T3540	−1,086	LmjF.35.3540	pre-rRNA-processing protein PNO1, putative
LmjF.36.T1620	−1,076	LmjF.36.1620	universal minicircle sequence binding protein (UMSBP2)
LmjF.28.T0720	−1,047	LmjF.28.0720	Sas10/Utp3/C1D family protein, putative
LmjF.34.T4290	−1,041	LmjF.34.4290	nucleolar protein family a, putative
LmjF.25.T0370	−1,025	LmjF.25.0370	Fcf2 pre-rRNA processing, putative
LmjF.35.T1820	−1,002	LmjF.35.1820	Eukaryotic rRNA processing protein EBP2, putative
LmjF.35.T2550	−1,000	LmjF.35.2550	Double RNA binding domain protein 9
**Protein translation**			
LmjF.35.T5040	−1,323	LmjF.35.5040	polyadenylate-binding protein 1 (PABP1)
LmjF.11.T0900	−1,155	LmjF.11.0900	60S ribosomal protein L24, putative
LmjF.03.T0980	−1,111	LmjF.03.0980	eukaryotic initiation factor 2a, putative
LmjF.09.T0970	−1,061	LmjF.09.0970	elongation factor-1 gamma (EF1G)
LmjF.36.T6980	−1,048	LmjF.36.6980	eukaryotic translation initiation factor 3 subunit 8, putative
LmjF.17.T1290	−1,015	LmjF.17.1290	translation initiation factor, putative
LmjF.16.T1600	−1,008	LmjF.16.1600	eukaryotic translation initiation factor 4 gamma, putative (EIF4G3)
**General metabolism**			
LmjF.21.T0845	−1,396	LmjF.21.0845	hypoxanthine-guanine phosphoribosyltransferase (HGPRT)
LmjF.30.T1890	−1,371	LmjF.30.1890	Adenylate kinase, nuclear
LmjF.23.T1580	−1,280	LmjF.23.1580	Nucleoside 2-deoxyribosyltransferase, putative
LmjF.35.T2160	−1,115	LmjF.35.2160	adenine aminohydrolase (AAH)
LmjF.12.T1270	−1,099	LmjF.12.1270	arginine N-methyltransferase-like protein
LmjF.36.T2360	−1,090	LmjF.36.2360	tyrosine aminotransferase (TAT)
LmjF.28.T1280	−1,085	LmjF.28.1280	phenylalanine-4-hydroxylase (PAH)
LmjF.13.T1680	−1,069	LmjF.13.1680	pyrroline-5-carboxylate reductase (P5CR)
LmjF.10.T0010	−1,003	LmjF.10.0010	fatty acid desaturase, putative
**Other functions**			
LmjF.07.T0800	−1,549	LmjF.07.0800	flavoprotein subunit-like protein
LmjF.36.T2570	−1,341	LmjF.36.2570	membrane-bound acid phosphatase precursor (MBAP)
LmjF.14.T1050	−1,257	LmjF.14.1050	COQ9, putative
LmjF.07.T0810	−1,250	LmjF.07.0810	cytochrome b5-like protein
LmjF.23.T0050	−1,140	LmjF.23.0050	cyclophilin 11, putative (CYP11)
LmjF.30.T2480	−1,131	LmjF.30.2480	heat shock 70-related protein 1, mitochondrial precursor, putative
LmjF.30.T0860	−1,124	LmjF.30.0860	surface protein amastin, putative
LmjF.13.T0090	−1,086	LmjF.13.0090	carboxypeptidase
LmjF.24.T1250	−1,021	LmjF.24.1250	amastin-like surface protein-like protein
**Hypothetical proteins**			
LmjF.06.T1290	−1,689	LmjF.06.1290	hypothetical protein, unknown function
LmjF.30.T0805	−1,542	LmjF.30.0805	hypothetical protein, conserved
LmjF.35.T4380	−1,313	LmjF.35.4380	hypothetical protein, conserved
LmjF.29.T0890	−1,304	LmjF.29.0890	Eukaryotic protein of unknown function (DUF914), putative
LmjF.32.T2940	−1,187	LmjF.32.2940	hypothetical protein, conserved
LmjF.29.T1870	−1,122	LmjF.29.1870	hypothetical protein, conserved
LmjF.30.T0090	−1,088	LmjF.30.0090	hypothetical protein, conserved
**Transcripts lacking protein-coding annotation**			
LmjF.10.T0032	−2,476	—	unknown (ORF coding for 59 amino acids)
LmjF.22.T0755	−1,823	—	unknown
LmjF.05.T1214	−1,774	—	unknown
LmjF.34.T2532	−1,450	—	unknown
LmjF.33.T0602	−1,383	—	unknown (ORF coding for 99 amino acids)
LmjF.24.T2345	−1,359	—	unknown (ORF coding for 78 amino acids)
LmjF.35.T4017	−1,327	—	unknown
LmjF.29.T0613	−1,227	—	unknown
LmjF.15.T1575	−1,186	—	unknown
LmjF.14.T1115	−1,170	—	unknown
LmjF.33.T0285-snoRNAs	−1,167	LmjF.33.snoRNA0124-122	Several C/D snoRNAs and H/ACA-like snoRNAs
LmjF.02.T0747	−1,150	—	unknown
LmjF.21.T1569.5	−1,102	—	Unknown (ORF coding for 87 amino acids)
LmjF.36.T4252	−1,093	—	Unknown (ORF coding for 120 amino acids)
LmjF.28.T3032	−1,025	—	Unknown (ORF coding for 257 amino acids; see Fig. 6)

^a^Transcript identifier (ID) according to Rastrojo *et al*.^34^. Additional information available at Leish-ESP server (http://leish-esp.cbm.uam.es/).

^b^Gene ID according to GeneDB database.

^c^Hypothetical protein: predicted bioinformatically; conserved: predicted protein of unknown function that is also annotated in *T. brucei* and/or *T. cruzi* genome (GeneDB.org); unknown: non-demonstrated protein-coding function.

Data reporting differential expression for some of these transcripts were found in the literature, and they are discussed briefly. In our study, the most down-regulated transcript with functional annotation was LmjF.29.T0620, which encodes for a putative transporter of the ABC family, ABCA10^59^. Interestingly, when *L. donovani* promastigotes are incubated in amastigote differentiation conditions (37 °C and acidic medium), the abundance of this protein (LinJ.29.0640) was found to decrease^47^. Also, the transcript LmjF.06.T1290 (gene LmjF.06.1290) was found to be 2.3-fold more abundant in promastigotes than in amastigotes^24^. Similarly, the level of the orthologue transcript in *L. infantum* (LinJ.06.1350) was found to be 1.7-fold higher in the promastigote form than in the amastigote stage^25^.

The protein encoded by transcript LmjF.36.T1640, named universal minicircle sequence binding protein (UMSBP), was found to be down-regulated when *L. donovani* promastigotes were incubated in amastigote differentiation conditions^47^. The cellular function of UMSBP has been studied in *Trypanosoma brucei* by RNAi experiments, revealing a key role in kDNA replication initiation and segregation as well as in mitochondrial and nuclear division^60^. Two more variants of this protein, UMSBP1 and UMSBP2, are encoded by transcripts LmjF.36.T1610 and LmjF.36.T1620, whose levels also experienced a 2.2-fold decrease (Table 3).

It is remarkable that three of the down-regulated transcripts encode for putative pteridine transporters (LmjF.06.T1260, LmjF.10.T0020 and LmjF.10.T1310). In agreement with our data, previous studies showed that transcript levels for pteridin transporters are more abundant in *L. major* promastigotes than in amastigotes^24,25^, even though the converse was observed in *L. infantum*^51^.

Another group of down-regulated transcripts were those coding for amino acids transporters: LmjF.31.T0880, LmjF.27.T1580, LmjF.31.T0340, LmjF.31.T0350, LmjF.35.T5350 and LmjF.31.T0580. In agreement with these results is the observation that the protein (aATP11), encoded by the gene LinJ.31.0360 (orthologous to genes LmjF.31.0340, LmjF.31.0350 and LmjF.31.0580), experienced a quick decrease when *L. donovani* promastigotes were incubated in amastigote differentiation conditions^47^.

There are eight down-regulated transcripts encoding proteins that may be associated with RNA metabolism: LmjF.07.T0870, LmjF.35.T2550, LmjF.32.T0840, LmjF.35.T3540, LmjF.28.T0720, LmjF.34.T4290, LmjF.25.T0370, and LmjF.35.T1820. In a previous study, transcript LmjF.32.T2550, which encodes for an RNA binding protein, was found to be more abundant in *L. major* promastigotes than in amastigotes^25^. Transcript LmjF.32.T0840 encodes for the orthologue to the *T. brucei* RNA-binding protein DRDB18^61^ and, interestingly, the orthologue protein in *L. donovani* (LinJ.32.0890) experienced a decrease in abundance after incubation of promastigotes at 37 °C in an axenic amastigote differentiation medium^47^. The transcript LmjF.28.T0720 encodes for a member of the Sas10/Utp3/C1D family, and the orthologous protein in *L. donovani* (LinJ.28.0770) was identified as down-regulated after incubation of promastigotes at 37 °C and acidic medium^47^.

Another set of transcripts that showed a temperature-dependent decrease in their steady-state levels can be grouped as translational regulatory factors: LmjF.35.T5040, LmjF.35.T3100, LmjF.03.T0980, LmjF.09.T0970, LmjF.36.T6980, LmjF.17.T1290 and LmjF.16.T1600. In previous studies, transcript LmjF.35.T5040 (coding for the poly-A binding protein 1, PABP1) was found to be more abundant in promastigotes than in amastigotes using oligonucleotide microarrays^24^. Also, the levels of proteins encoded by transcripts LmjF.35.T5040 (PABP1), LmjF.35.T3100 (RNA helicase of the DEAD-box family, DED1), LmjF.03.T0980 (eukaryotic initiation factor 2 alpha, eIF2α), LmjF.09.T0970 (elongation factor 1 gamma, EF1G), LmjF.36.T6980 (eukaryotic translation initiation factor 3 subunit 8, eIF3c), LmjF.17.T1290 (eIF3b), and LmjF.16.T1600 (eIF4G3) decreased when *L. donovani* promastigotes were incubated at 37 °C in an axenic amastigote growth medium^47^. In addition, protein PABP1 and eIF3b were found to be more abundant in membrane enriched-fractions prepared from *L. infantum* promastigotes than in those derived from axenic amastigotes^50^. The DED1 proteins are associated with translation initiation in eukaryotes, and they have been studied in *Leishmania amazonensis*^62^. In the *Leishmania* genome, there are two paralogs, DED1-1 and DED1-2, that show differential expression during parasite development; thus, the protein LeishDED1-2 (encoded in transcript LmjF.35.T3100) is more abundant in promastigotes, being almost undetectable in the amastigote stage, whereas expression of the paralog LeishDED1-1 increases in axenic amastigotes^62^. Overall, these data support the notion that translation machinery would be tightly regulated by environmental changes, acting as a regulatory factor for stage differentiation in *Leishmania*. This is in agreement with the study by Lahav and co-workers^63^ in which a down-regulation of the translation machinery, mediated largely at the level of mRNA stability, takes place in *L. donovani* during the axenic promatigote-to-amastigote differentiation.

Among transcripts down-regulated at 37 °C (Table 3), the presence of two transcripts coding for amastins was somewhat unexpected, taking into account that amastins are known to be over-expressed during the amastigote stage^54^, and amastin transcripts result highly up-regulated during heat shock^64^. In fact, in this study, we have found that the steady-state levels of several amastin transcripts increased after incubation at 37 °C of *Leishmania* promastigotes (see above and Table 2). Nevertheless, our data indicated that the levels for transcripts LmjF.30.T0860 and LmjF.24.T1250 clearly decreased after heat shock. Interestingly, in a previous report, using cDNA microarrays, transcript LmjF.30.T0860 was found to be more abundant in *L. major* promastigotes than in amastigotes^32^. Furthermore, the orthologous protein in *L. infantum* (LinJ.30.0920) was found to be membrane-enriched fractions of promastigotes^50^. Evolutionary analyses have shown that the amastin family experienced a substantial expansion directly associated with the origin of the genus *Leishmania*, probably some amastin genes evolved to perform novel functions in other phases of the parasite life cycle^65^.

Coincidental results, considering higher expression in promastigotes regarding amastigotes or amastigote-like forms, were also observed for transcripts LmjF.07.T0800, LmjF.29.T0890, LmjF.35.T2160, LmjF.36.T2360, LmjF.13.T0090, and LmjF.13.T1680^25^. Protein down-regulation after incubation of *L. donovani* promastigotes under axenic amastigote growth conditions^47^ was observed for the orthologous to the proteins encoded by the transcripts LmjF.21.T0845 (LinJ.21.0980), LmjF.30.T1890 (LinJ.30.1880), LmjF.35.T4380 (LinJ.35.4450), LmjF.23.T1580 (LinJ.23.1980), LmjF.11.T0900 (LinJ.11.0900), LmjF.23.T0050 (LinJ.23.0060), LmjF.30.T2480 (LinJ.30.2480), LmjF.35.T2160 (LinJ.35.2200), LmjF.12.T1270 (LinJ.12.0850), LmjF.36.T3070 (LinJ.36.3220), LmjF.36.T2360 (LinJ.36.2490), LmjF.13.T0090 (LinJ.13.0090), LmjF.13.T1680 (LinJ.13.1420), and LmjF.10.T0010 (LinJ.10.1460).

**Figure 6:**
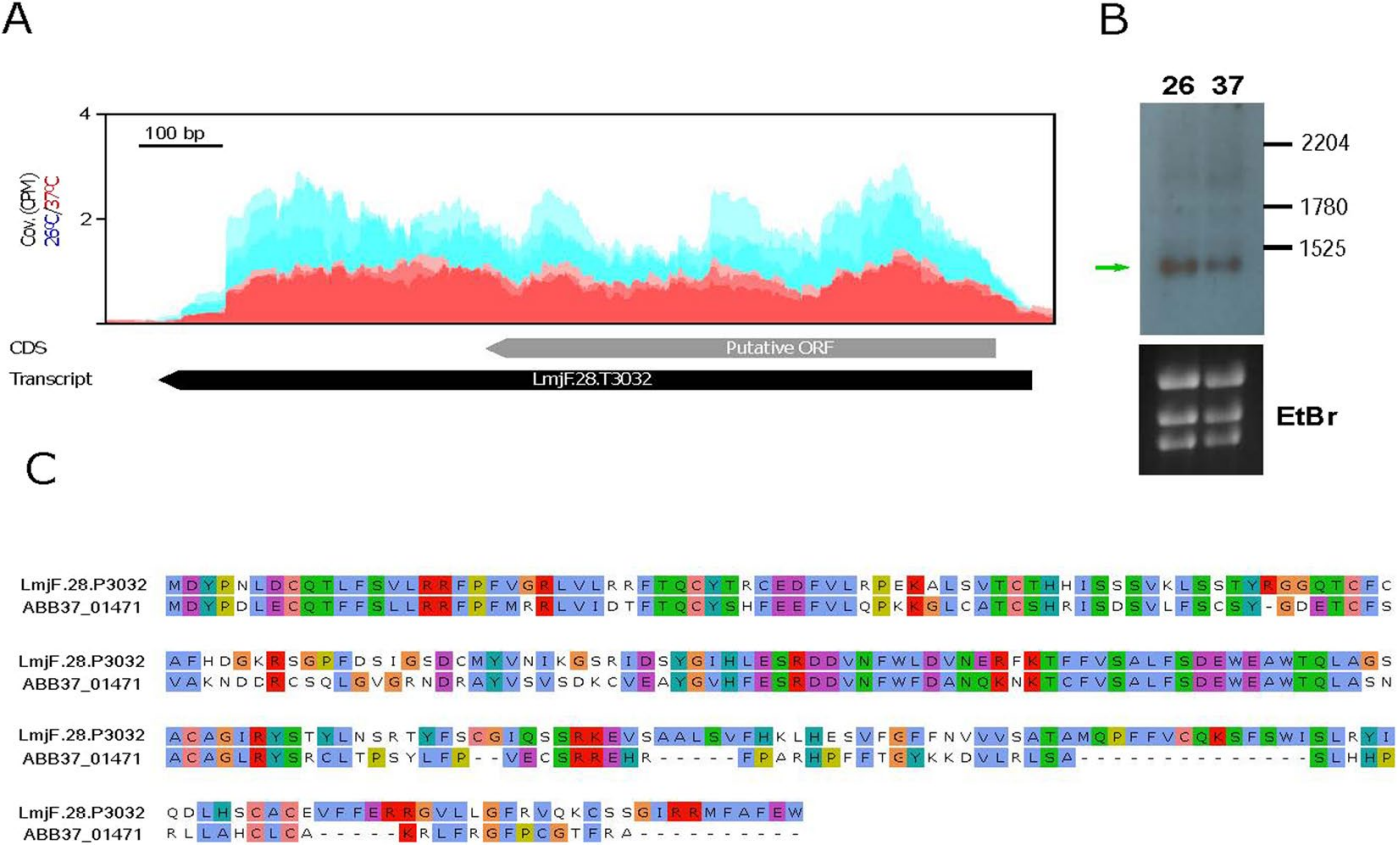
Temperature-dependent expression of transcript LmjF.28.T3032, which encodes for a previously non-annotated protein. (**A**) Alignment of the RNA-seq reads derived from RNA samples (three biological replicates) isolated from either promastigotes growth at 26 °C (blue) or heat shocked at 37 °C (red). The location of the transcript is indicated by a black arrow, whereas the location of the putative ORF is shown by a grey arrow. Coverage (Cov.) is expressed as counts per million of reads (CPM). (**B**) Analysis by Northern blotting of transcript LmjF.28.T3032 in RNA samples derived from parasites incubated at 26 or 37 °C. The sizes (in nucleotides) of the *L. major* rRNA molecules^72^ used as molecular markers are indicated. Bottom, ethidium bromide (EtBr) staining of the gel, before membrane transfer and hybridization. Uncropped images shown in Supplementary Figure 1. (**C**) Sequence alignment between the protein ABB37_01471 of *L. pyrrhocoris* and the putative protein encoded in transcript LmjF.28.T3032 (LmjF.28.P3032).

In addition, we detected several transcripts, currently lacking of any annotation in the *L. major* (Friedlin) databases, that were significantly down-regulated by heat shock. In fact, the three transcripts with the highest FC values are within this category (Table 3). An analysis of ORFs in these transcripts indicated that some of them might be protein-coding mRNAs. Thus, transcript LmjF.10.T0032 (FC = 5.7) contains an ORF coding for a polypeptide of 59 amino acids, which is 100% identical in sequence and length to a hypothetical protein annotated in the genomes of the *L. major* strains LV39 and SD75 (TriTrypDB.org). Also, transcript LmjF.28.T3032 has an ORF predicting a protein of 257 amino acids; this putative protein is 100% identical to the protein entry ID: LMARLEM2494_000007900.1-p1 (TriTrypDB database), annotated in the genome of the *Leishmania* sp MAR_LEM2494 strain. Moreover, the predicted protein has 54% of sequence identity (69% of sequence similarity; Fig. 6) with protein ABB37_01471 (UniProtKB database) from *Leptomonas pyrrhocoris*, a monoxenous trypanosomatid evolutionary related to the genus *Leishmania*^66^. Other transcripts having potential protein-coding function are LmjF.33.T0602 (encoding for a 99 amino acids long polypeptide), LmjF.24.T2345 (polypeptide of 78 aas), LmjF.21.T1569.5 (87 aas), and LmjF.36.T4252 (120 aas); however, the predicted polypeptides do not share any significant sequence homology with proteins deposited in databases. Therefore, specific experiments addressing the existence of these hypothetical proteins would be needed before to ascribe a protein coding function for these transcripts.

### Alternative *trans-*splicing as an additional layout for regulating gene expression in *Leishmania*

It was previously reported^34^ that around 50% of the *L. major* annotated genes may originate different transcripts differing in the SL addition site (SAS). This leads to the existence of transcripts with different length in their 5′-UTRs and sometimes also in their ORF, increasing in turn the coding capacity and the regulatory possibilities for a given gene. After mapping and analysing the *trans-*splicing addition sites in the different *L. major* transcripts using RNA-seq data in the two experimental conditions assayed in this work, new features and possible functions for the *trans-*splicing were glimpsed.

**Figure 7:**
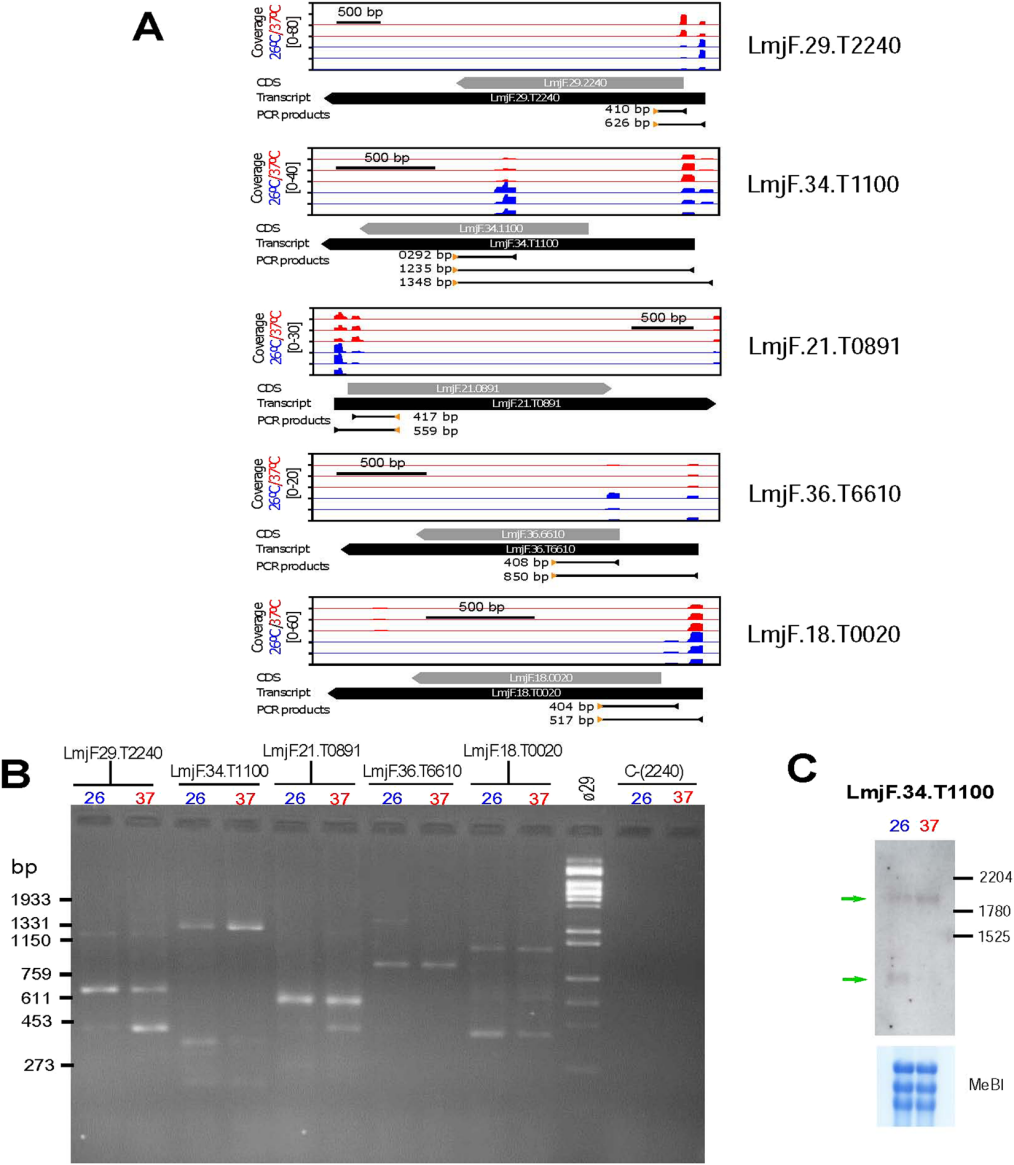
Changes in the selection of SL-addition sites (SASs) associated with the temperature treatment in *L. major* promastigotes. (**A**) Schematic representation of the SASs used for *trans*-splicing of transcripts LmjF.29.T2240, LmjF.34.T1100, LmjF.21.T0891, LmjF.36.T6610 and LmjF.18.T0020 as determined by position of SL-containing RNA-seq reads for each one of the three experimental replicates from either promastigotes grown at 26 °C (blue) or promastigotes incubated for 2 h at 37 °C (red). For each transcript, the relative coverage is shown according to the scale indicated. Below each graph, the position of the CDS (grey arrow), transcript (black arrow) and the size and position of the expected PCR amplification products are shown. (**B**) PCR amplification of cDNA synthetized from RNA samples of parasites incubated at either 26 °C or 37 °C. For each transcript, a specific oligonucleotide and a common SL-oligonucleotide were used (oligonucleotide sequences are indicated in the Methods section). The negative control, C-(2240), consisted of the amplification from the RNA samples (without retrotranscription step) using the LmjF.29.T2240-specific oligonucleotide and the SL-oligonuclotide. *Hin*dIII-digested DNA of bacteriophage Φ29 was used as size marker (lane Φ29), and the size of relevant bands is indicated on the left. (**C**) Analysis by Northern blotting of transcripts derived from gene LmjF.28.3032 in RNA samples derived from parasites incubated at 26 or 37 °C. Bottom, methylene blue (MeBl) staining of the membrane used for hybridization. Uncropped images shown in Supplementary Figure 2.

Firstly, a global analysis of the RNA-seq reads derived from the two experimental conditions (26 °C and 37 °C) indicated that 8,069 out of the 10,285 genes annotated in the *L. major* genome^34^ (http://leish-esp.cbm.uam.es) contains two or more SL addition sites (see Supplementary Dataset 2). Based on the relative frequencies of SAS usage for the two conditions, it was found that after the heat shock treatment of *L. major* promastigotes, a change in the preferential SAS occurred in 566 genes. For 152 out of these 566 genes, the distance between the SAS used at normal growth and the one used at heat shock conditions was larger than 100 nucleotides. Figure 7 shows examples of genes in which such a change in the SAS occurred. RT-PCR validation was done by amplification of cDNA using a common forward oligonucleotide (derived from the mini-exon sequence) and a specific reverse oligonucleotide for each selected gene (see Fig. 7A for design details). Amplification products were analysed by agarose gel electrophoresis (Fig. 7B), and the results were in agreement with the expected sizes of the transcripts according with the preferential temperature-SAS usage as determined by mapping of SL-containing RNA-seq reads (Fig. 7A). Only for gene LmjF.18.0020, an additional amplification product of about 1-kb was detected in both samples, suggesting the existence of an additional SAS upstream of the expected ones.

Among the analysed transcripts, the SAS usage in transcripts derived from gene LmjF.34.1100 is really striking. According to the RNA-seq data, at 26 °C, a significant fraction of the transcripts derived from gene LmjF.34.1100 would be spliced within the ORF and, therefore, a truncated version of the encoded protein would be generated. However, at 37 °C, the main SAS would take place upstream of the ORF (Fig. 7A). The RT-PCR experiments confirmed the change in SAS after heat shock treatment; thus, a 292-bp amplicon was visible only for the RNA sample obtained from promastigotes grown at 26 °C (Fig. 7B). To further verify this observation, we analysed by Northern blotting the transcripts derived from this gene in both conditions. As shown in Fig. 7C, the smallest transcript is only observed at 26 °C whereas the intensity of the complete transcript increased at 37 °C. The predicted ORF of gene LmjF.34.1100 encodes for a hypothetical protein of 385 amino acids, which has not ascribed any function but it is conserved in other trypanosomatids like *T. brucei* and *T. cruzi*. However, the addition of the SL into the ORF sequence occurring at 26 °C would generate a shorter transcript, coding for a polypeptide of 158 amino acids in length, but having different sequence to the protein encoded from the full ORF as they are translated following different frames. Work is ongoing to analyse whether this short polypeptide is really produced in the parasite and to look for a possible functional relevance of this SAS change associated with heat shock.

## Conclusions

The heat shock response, a universal mechanism aimed to counteract deleterious effects on cellular homeostasis promoted by a sudden increase in the ambient temperature, is classically used as a model system to study gene expression^67^. Nevertheless, given that *Leishmania* parasites are subjected to a natural heat shock during infection of mammalian hosts, in addition to a protective role for parasite survival, the heat shock proteins may be involved in the molecular mechanisms leading to parasite differentiation. In this work, we have analysed by RNA-seq the effect of a moderate heat treatment (2 h at 37 °C) on the *L. major* transcriptome (promastigote stage). Significant changes in the mRNA levels were observed in 3969 out of the 10700 transcripts that are currently annotated for this species; of these, 1,866 were up-regulated and 2,103 down-regulated. In general, based on coincidental results found in articles studying differential gene expression at RNA and/or protein levels during axenic promastigote-to-amastigote differentiation, it can be concluded that heat shock leads to the up-regulation of amastigote-specific transcripts whereas promastigote-specific transcripts results down-regulated. However, this is not a demonstration that heat shock is the inducer of parasite differentiation.

Similar to alternative *cis-*splicing, which is likely to be the most important engine driving the diversity of proteins existing in any given cell^68^, alternative *trans-*splicing in *Leishmania* and related trypanosomatids seems to be playing a relevant role in modelling regulatory networks and even the proteome compendium at the different developmental stages. As shown here, RNA-seq is a valuable tool for identification of changes in the SAS usage and thereby for defining alternative *trans-*splicing events and uncovering novel protein products. Moreover, although many alternative SAS do not affect the encoded proteins, they generate different 5′ UTRs that may influence the stability, translation and cellular location of mRNAs.

Finally, this work claims the use of detailed transcriptome annotations as a crucial step to be accomplished before analysing differential gene expression at a genome-whole scale. Most of the studies addressing differential gene expression in *Leishmania* by using RNA-seq consider only ORFs for reads mapping. However, as illustrated in Figs 4 and 5, caution should be taken when analysing gene expression data from gene families sharing identical ORFs but differing in the UTRs.

## Materials and Methods

### Leishmania culture and RNA isolation

Promastigotes of *L. major* Friedlin strain (MHOM/IL/80/Friedlin; clone V1) were cultured at 26 °C in M199 medium supplemented with foetal bovine serum (10%), HEPES (40 mM; pH 7.4), adenine (0.1 mM), hemin (10 µg/ml), biotin (1 µg/ml) biopterin (2 ng/ml), penicillin G (100 U/ml) and streptomycin sulphate (0.1 mg/mL). This strain was provided by Dr. Javier Moreno, Instituto de Salud Carlos III (Madrid, Spain), a WHO reference centre for leishmaniasis. Promastigote cultures (50 ml) were seeded at 1 × 10^6^ cells/ml and incubated at 26 °C; when the cultures arose the logarithmic-phase (days 2–3), they were split in two subcultures (25 ml each) that were incubated for 2 h either at 26 °C or 37 °C. Three different cultures (biological replicates) were processed independently. The densities (cells/ml) of the cultures just before the temperature incubation were 6.1 × 10^6^ (replicate 1), 7.8 × 10^6^ (replicate 2) and 18 × 10^6^ (replicate 3).

After incubation, parasites were harvested by centrifugation, and the pellet suspended in 1 ml of Trizol (TRI Reagent, Sigma-Aldrich, product No. T9424). Manufacturer’s instructions were followed. RNA samples were suspended in DEPC-treated water, and their concentrations were determined using the Nanodrop ND-1000 (Thermo Scientific); all samples showed A_260_/A_280_ ratios higher than 2.0. In addition, RNA integrity was checked in a bioanalyzer (Agilent 2100).

### Next-generation sequencing

Library preparations (mRNA and DNA) and sequencing were performed by Centro de Análisis Genómico (CNAG, Spain). Sequencing was done using Illumina HiSeq 2000 technology. From total RNA, purification of the poly-A^+^ fraction was conducted before RNA fragmentation for library construction. Paired reads of 75 nucleotides were generated, and the number of reads obtained for each sample is indicated in Table 1. Sequence quality metrics were assessed using FastQC (https://www.bioinformatics.babraham.ac.uk/projects/fastqc/).

### Mapping of RNA-seq to reference genome and transcriptome

Reads were mapped to the *L. major* (Friedlin strain) genome^37^, which can be downloaded at Leish-ESP web page (http://leish-esp.cbm.uam.es/). Alignments were done by Bowtie2^69^ and reads having 96% (or higher) sequence identity were selected for further analyses (Table 1). Reads that mapped to multiple loci were kept but only a single alignment (randomly distributed) was reported for each read (default, −k 1).

*L. major* (Friedlin strain) transcriptome was delineated as described elsewhere^34^, and the updated version (v1.2/2017), available at Leish-ESP server, was used. Afterwards, the HTSeq program^70^ was used for counting the reads overlapping with the 10,700 annotated transcripts. These sets of values (3 biological replicates for each growth condition) were used for differential expression analysis by the DESeq2 program^71^, using an adjusted p-value < 0.05 as statistically significant to consider that a transcript showed differential expression between the two conditions assayed. According to the number of reads aligned to the rRNA transcripts, it was calculated that they represent 0,91% and 0,77% of the total mapped reads in the RNA-seq data derived from promastigotes incubated at 26 °C or 37 °C, respectively.

### Determination of changes in the preferential SL addition sites (SAS) from RNA-seq data

Unmapped reads were used for searching SL-containing reads. Firstly, unmapped reads were quality filtered using Prinseq (mean quality ≥25). Then, an in-house Python script was used for searching SL sequences in the reads. Only reads containing at least 8 nucleotides of the SL were considered for further analysis. After trimming out the SL sequence, the rest of the reads were mapped back to the reference genome in order to define genomic coordinates (and chromosome location) of the identified SL addition sites (SAS). Upstream genomic regions were tested for SL-like coding sequences to avoid potential false positive SL identification (<4 mismatches/39 nucleotides). Additionally, only concordant mapped paired-reads were considered as true positive SL-containing reads. The procedure was repeated for each one of the six RNA-seq samples (three from parasites cultured at 26 °C and another three from parasites cultured at 37 °C). Counts were normalized according to the total number of SL-containing reads found in each RNA-seq sample, using the tool edgeR and the function CPM (counts per million). Only SAS with CPM values higher than 1 in at least 3 out of the six samples were considered. As a result, 27,622 different SAS were identified and used for further analysis.

Then, SAS were assigned to the annotated transcripts^34^. A SAS was consider to belong to a transcript if it is located between the beginning of the transcript (or upstream of it, with the limit of 20% of the length of the transcript) and the end of the transcript (minus 20% of the length of the transcript and separated at least 300 bp from the downstream transcript). When two or more SAS were present in a transcript, the principal and secondary SAS were defined according to their cumulative number of reads mapping a given SAS. Then, we calculated the log_2_-ratio between principal and secondary SAS for each transcript at each condition (26 °C and 37 °C), considering cumulative normalized counts of the three replicates per experimental condition. Among the transcripts showing differential SAS usage between both growth conditions (see Supplementary Dataset 2), we selected for further analysis those transcripts having principal and secondary SAS separated 100 or more nucleotides each other.

### Quantitative real-time PCR (qPCR) analysis

After RNA purification by the TRI Reagent manufacturer’ protocol (Sigma-Aldrich) from new experimental replicates (other than those used for RNA-seq), RNA samples were treated with the TURBO DNA-free Kit (Thermo Thermo Fisher Scientific) for the digestion of DNA traces (the procedure included a post-digestion step for the removal of the enzyme and divalent cations). Afterwards, RNA samples were reverse transcribed using the oligo-(dT) primer and the ThermoScript RT-PCR system (Invitrogen). Products were diluted in water and amplified by quantitative PCR using the specific primers: 5′-AGTCCAGTTG TCCGCCATC-3′ and 5′-CAATAAAGGC TCGCGTGGAC-3′ for transcript LmjF.02.T0460; 5′-CACCAAAATT TGCTGCGTC-3′ and 5′-AAGCGGATTG ACAAGGACAG-3′ for transcript LmjF.32.T2260; 5′-TGCTTGGTCT CCGATCCTTG-3′ and 5′-CCCTGTTGTC AAACCACACG-3′ for transcript LmjF.06.T1260; 5′-GGTTACCTCC ATGAGTGACG-3′ and 5′-GAAAGCGGGC TACTGCAAG-3′ for transcript LmjF.36.T3000; 5′-CTTGTGGGAG CTAGGGATG-3′ and 5′-TGTCGACTCC GCATAGTGC-3′ for transcript LmjF.16.T1650.

Quantitative real-time PCR (qPCR) was performed in an ABI Prism 7900HT Detection System (Applied Biosystems) with Power SYBR Green Master Mix (Applied Biosystems) at the Genomics and NGS Facility (CBMSO). Standard curves were prepared from five serial, five-fold dilutions of genomic DNA that were amplified using the same conditions and reaction mixture used for amplification of the samples. All qPCRs were implemented in duplicates and in two independent samples accepting only those experiments with parameters of the standard curve within −3.3 ± 10% and ≥0.99 for the slope and R^2^ values, respectively. In parallel, qPCR assays were done directly from RNA samples to exclude the presence of background DNA in the samples (negative control). Data analysis was performed using the ABI Prism 7900HT SDS Software (v. 2.4).

### Northern blotting

Total RNA (8 µg per sample) was denatured for 10 min at 65 °C in loading buffer and separated on a 1.5% agarose formaldehyde/MOPS gel for 16 hours at 45V. The RNA was then transferred onto a positively charged nylon membrane (Roche Diagnostics GmbH) by capillary transfer method using 20× SSC buffer. The membrane was crosslinked on a GeneLinker at 120 mJ, stained with methylene blue, prehybridized in DIG Easy Hyb Hybridization solution (Roche Diagnostics GmbH) for 1 hour at 50 °C and hybridized for 16 hours at 50 °C with DIG-labelled DNA probes diluted in DIG Easy Hyb solution. Stringency washes and chemiluminescent detection were performed following the manufacturer’s instructions.

DNA sequences to be used as probes were PCR amplified, cloned into pNZY28 plasmid (NZYTech) and sequenced. For amplification of the probe specific for LmjF.28.3032-derived transcripts, *L. major* genomic DNA and the following oligonucleotides were used: forward, 5′-GGATCCATGGACTATCCGAACTTAGAC-3′; reverse, 5′-CTGCAGCTCACCATTCGAATGCAAAC-3′. The LmjF.34.T1100 probe was generated by amplification of *L. major* cDNA (see below) with the oligonucleotides: forward, 5′-AGCTGCTATATAAGTATCAGTTTCTGTAC-3′; reverse, 5′-TGATGTCTGCTACAGAGAC-3′. DIG-labelled DNA probes were generated by the PCR DIG Labelling kit (Roche Diagnostics GmbH) following the manufacturer’s instructions. Finally, the DIG-labelled products were purified using the FavorPrep Gel/PCR purification mini kit (Favorgen Biotech Corp, Taiwan).

### Experimental analysis of changes in the preferential SL addition sites during heat shock

RNA samples from *L. major* promastigotes either grew at normal temperature (26 °C) or further incubated for 2 h at 37 °C were obtained as described above. RNA was retrotranscribed into cDNA using oligo-dT and the ThermoScript RT-PCR system (Invitrogen). Afterwards, cDNA aliquots from each treatment were PCR amplified using a common forward oligonucleotide, CJM3 (5′-GCTATATAAG TATCAGTTTC TGTAC-3′), which contains part of the SL sequence, and the following specific oligonucleotides (reverse primers): LmjF.29.T2240_Rv, 5′-AAGTTCACCT GGTAGAAGC-3′; LmjF.34.T1100_Rv, 5′-TGATGTCTGC TACAGAGAC-3′; LmjF.21.T0891_Rv, 5′-TGAAGAACGT CCTCAGTC-3′; LmjF.36.T6610_Rv, 5′-AGTACACAAG CTCACTTAGC-3′; LmjF.18.T0020_Rv, 5′-CTTCAGATTC TTCGTGTTG-3′.

## Supplementary information

Supplementary Information

Dataset 1

Dataset 2

Dateset 3

## Data Availability

The RNA-seq data reported in this study is freely available at the European Nucleotide Archive (ENA; http://www.ebi.ac.uk/ena/) under project number PRJEB27042.
