# Supplementary material for: Analysis by RNA-seq of transcriptomic changes elicited by heat shock in Leishmania major
Source: Sci Rep. 2019 May 6;9:6919. doi: 10.1038/s41598-019-43354-9 (PMC6502937; doi:10.1038/s41598-019-43354-9)
Supplement: Supplementary file 1 — Supplementary Information [file 41598_2019_43354_MOESM1_ESM.pdf]

SUPPLEMENTARY INFORMATION

Analysis by RNA-seq of transcriptomic changes elicited by heat shock in *Leishmania major*

Alberto Rastrojo, Laura Corvo, Rodrigo Lombraña, Jose C. Solana, Begoña Aguado, Jose M. Requena

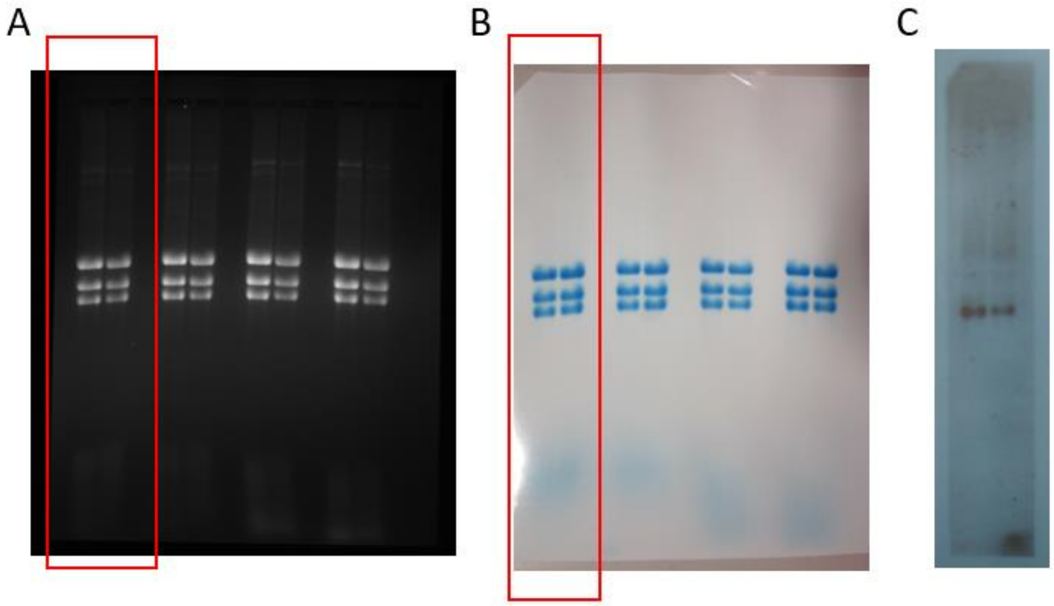

Supplementary Figure 1.
Four pairs of RNA samples (26º and 37ºC treatments) were size-separated on 1% (w/v) agarose-formaldehyde gel. Panel A, Ethidium bromide stained gel. Afterwards, the RNAs were electrophoresed onto a nylon membrane, and the membrane was soaked for 5 min at room temperature in a solution containing 0.5M sodium acetate pH 5.2 and 0.04% methylene blue (MeBl); the result is shown in panel B. The membrane was split into four equivalent pieces. The part of the membrane indicated by the red rectangle was used for detecting the transcripts derived from gene LmjF.28.3032. Panel C shows the autoradiograph obtained after hybridization and detection steps. Panel B of figure 5 was made from the autoradiograph (panel C) and the corresponding part of the membrane (stained with MeBl) shown in panel B.

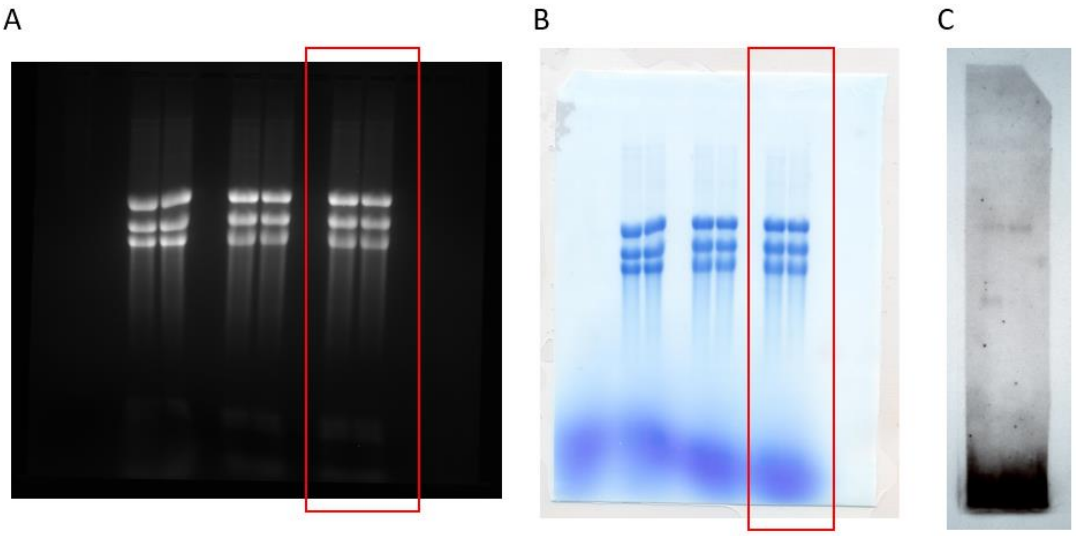

Supplementary Figure 2.
Three pairs of RNA samples (26º and 37ºC treatments) were size-separated on 1% (w/v) agarose-formaldehyde gel. Panel A, Ethidium bromide stained gel. Afterwards, the RNAs were electrophoresed onto a nylon membrane, and the membrane was soaked for 5 min at room temperature in a solution containing 0.5M sodium acetate pH 5.2 and 0.04% methylene blue (MeBl); the result is shown in panel B. The membrane was split into three equivalent pieces. The part of the membrane indicated by the red rectangle was used for detecting the transcripts derived from gene LmjF.34.1100. Panel C shows the autoradiograph obtained after hybridization and detection steps. Panel C of figure 7 was made from the autoradiograph (panel C) and the corresponding part of the membrane (stained with MeBl) shown in panel B.
